# Diabetes mellitus and health-related quality of life in long-term survivors of breast, colorectal or prostate cancer: a population-based prospective study

**DOI:** 10.1038/s41416-026-03463-5

**Published:** 2026-05-13

**Authors:** Keyi Yang, Melissa S. Y. Thong, Daniela Doege, Lena Koch-Gallenkamp, Linda Weisser, Heike Bertram, Andrea Eberle, Bernd Holleczek, Alice Nennecke, Annika Waldmann, Sylke Ruth Zeissig, Ron Pritzkuleit, Lina Jansen, Hermann Brenner, Volker Arndt

**Affiliations:** 1 Cancer Survivorship Outcomes and Epidemiology, German Cancer Research Center (DKFZ), Heidelberg, Germany; 2 Division of Clinical Epidemiology of Early Cancer Detection, DKFZ, Heidelberg, Germany; 3 Division of Clinical Epidemiology and Aging Research, DKFZ, Heidelberg, Germany; 4 Cancer Registry of North Rhine-Westphalia, Bochum, Germany; 5 Bremen Cancer Registry, Leibniz Institute for Prevention Research and Epidemiology—BIPS, Bremen, Germany; 6 Saarland Cancer Registry, Saarbrücken, Germany; 7 Hamburg Cancer Registry, Hamburg, Germany; 8 Institute for Social Medicine and Epidemiology, University of Lübeck, Lübeck, Germany; 9 Cancer Registry of Rhineland-Palatinate, Mainz, Germany; 10 Institute of Clinical Epidemiology and Biometry (ICE‑B), Julius Maximilian University of Würzburg, Würzburg, Germany; 11 Cancer Registry of Schleswig-Holstein, Lübeck, Germany; 12 Cancer Prevention Graduate School, DKFZ, Heidelberg, Germany; 13 Network Aging Research (NAR), University Heidelberg, Heidelberg, Germany

**Keywords:** Cancer epidemiology, Comorbidities

## Abstract

**Background:**

We examined the association between diabetes mellitus (DM) and health-related quality of life (HRQOL) in long-term cancer survivors (LTCS) 14–24 years post-diagnosis, and evaluated the role of DM in the HRQOL of cancer survivors and cancer-free controls.

**Methods:**

We analysed 6811 LTCS diagnosed with breast, colorectal, or prostate cancer between 1994 and 2004, surveyed in 2008–2011 and 2018–2019, and 1701 controls surveyed in 2013–2014. Multiple linear regression and linear mixed regression models were used to assess cross-sectional and prospective associations of DM and cancer with HRQOL, between participants with and without cancer, and between diabetics and non-diabetics.

**Results:**

At baseline, DM prevalence was 14%, and mean age of LTCS with DM was 72 years. LTCS with DM reported consistently poorer HRQOL than non-diabetic counterparts at baseline, with some HRQOL dimensions showing stronger associations with DM than with cancer in cross-sectional analysis. Although cancer generally showed a stronger association with poorer HRQOL than DM, DM was more strongly associated with worse physical functioning, global health status, pain, and appetite loss. The longitudinal perspective revealed similar associations between DM and HRQOL as the cross-sectional perspective for all scales.

**Conclusion:**

The findings highlight the need for comprehensive care strategies that address both cancer and DM to enhance health outcomes in diabetic LTCS.

## Introduction

Cancer remains a significant health issue in today’s healthcare landscape. In Europe, it is estimated that cancer survivors, i.e. those living with or beyond a diagnosis of cancer, currently represent around 5% of the population, with over 60% having survived five or more years after diagnosis, classifying them as long-term cancer survivors (LTCS) [1]. In Germany, nearly 5 million men and women were living with or after cancer at the end of 2020 [1]. Breast, prostate, and colorectal cancers collectively constitute over 44% of all cancer cases in female or male cancer survivors. Over 70% of breast cancer survivors, as well as around 65% of colorectal and prostate cancer survivors can be classified as LTCS [2].

Diabetes mellitus (DM) prevalence was 10% in Germany in 2021 [3], with higher rates in cancer survivors compared to non-cancer controls. The prevalence of DM can be close to 30% in survivors with breast, colorectal, or prostate cancer [4–9]. This is a result of multiple factors. Cancer survivors may be at a higher risk of DM than the non-cancer population due to biological interactions between cancer and DM, as well as cancer treatment such as radiotherapy, glucocorticoids, and hormone therapy [10–15]. Other factors include shared risk factors (such as obesity and reduced physical activity) and potentially increased detection rates due to frequent medical checks in cancer survivors during the aftercare.

Among cancer survivors, those with prevalent DM might have worse survival [16–19], poorer HRQOL [20–23], and less positive change in HRQOL over time [24, 25] than non-DM survivors. Nevertheless, such studies often have limitations such as cross-sectional designs, small sample sizes, or a focus on specific treatment methods. We are interested in understanding how DM and cancer are independently associated with HRQOL in LTCS, and whether DM is more strongly associated with HRQOL in LTCS compared to those without cancer. Only few studies have examined the combined association of cancer and DM with HRQOL [26–28]. It appears that cancer may be more strongly associated with lower HRQOL than DM, both mentally and physically [28]. This could be due to the effects of cancer and its treatments, such as surgery, chemotherapy and radiotherapy, as well as the perception of cancer as a more immediate threat to life compared to DM. However, it remains unclear whether this association persists or changes after living with or beyond cancer for an extended period in contrast to persisting and potentially aggravating limitations in patients with a chronic disease like DM.

Both the number of cancer and DM survivors is increasing in developed countries due to demographic ageing, improved prognosis, and unfavourable trends in obesity and sedentary lifestyle [29]. An improved understanding of the association between DM and long-term HRQOL in cancer survivors could help clinicians and survivors conduct better DM management. In this study, we aimed to assess both cross-sectional and prospective associations between DM and HRQOL in LTCS of breast, colorectal, or prostate cancer up to 24 years past after cancer diagnosis. Additionally, we aimed to assess the extent to which cancer and DM are associated with HRQOL, and whether the association between DM and HRQOL differs between cancer survivors and cancer-free controls.

## Materials and methods

### Study population

**Fig. 1:**
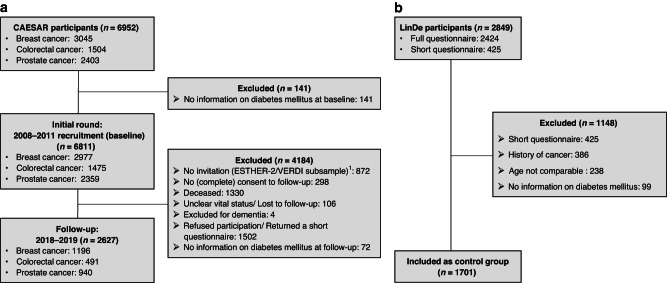
Flowcharts of the study sample inclusion. **a** CAESAR study (initial and follow-up surveys). **b** LinDe study. Footnote: ^1^ No follow follow-up was possible for participants from two sub-cohorts for which consent to store names and contact details had expired in 2010.

LTCS were selected from the population-based “Cancer Survivorship—A Multi-regional Population-Based Study” cohort (CAESAR). Details on the data collection have been reported previously [30, 31]. In brief, LTCS diagnosed between 1994 and 2004 were recruited via six population-based cancer registries in Germany in 2008–2011 (baseline). Following vital status checks, those alive and agreed to further follow-up were re-contacted in 2018–2019 (follow-up). Questionnaires were sent to cancer survivors who met the following inclusion criteria at baseline: age at diagnosis between 20 and 75 years; histologically confirmed cases of breast, colorectal, or prostate cancer. We received completed questionnaires from 6952 survivors at baseline, of whom 6811 provided information on DM. Among these participants, 2,627 individuals participated and provided information on DM at follow-up (Fig. 1).

Cancer-free population controls were selected from the LinDe (Lebensqualität in Deutschland—Quality of Life in Germany) study, a cross-sectional nationwide study. Details on the study population and procedures have been previously published [30]. Participants aged at least 18 years were randomly selected after age stratification and received a questionnaire by mail between 2013 and 2014. In total, 2849 LinDe participants returned a questionnaire, and 1701 of these, who completed the full-length questionnaire, were cancer-free, of comparable age, and provided adequate information on DM, were included as controls for this analysis (Fig. 1).

The studies were approved by the ethics committee of the Medical Faculty of Heidelberg University (CAESAR: S438/2008; LinDe: S499/2012). Additionally, as a multi-regional study, the CAESAR study received approval from all local ethics committees responsible for each participating cancer registry.

### Measurement of HRQOL

The European Organization for Research and Treatment of Cancer Quality of Life Questionnaire Core 30 (EORTC QLQ-C30) was used to measure HRQOL for participants in both CAESAR and LinDe studies [32]. This 30-item questionnaire contains five functional scales (physical, role, emotional, cognitive, and social), nine items/scales on common symptoms in cancer patients and financial difficulties, and a global health/quality-of-life (QoL) scale. For the two items in the global health/QoL scale, answers range from 1 (very poor) to 7 (excellent). For all other items/scales, respondents can choose from 1 (not at all) to 4 (very much). Scores are linearly transformed to a 0-100 scale according to the EORTC scoring manual [33]. Higher scores on the global health/QoL and functioning scales indicate better functioning, while higher scores on symptom and financial difficulties scales indicate greater burden. A summary score, excluding the global health/QoL and financial difficulties scales, was derived from the arithmetic mean of 13 out of the 15 scales [34].

### Assessment of chronic conditions (including DM)

Participants were asked whether they had DM with response options of Yes, No, or Unknown, in both the CAESAR and LinDe questionnaires. This question did not differentiate between different types of DM. In the CAESAR study, we also assessed, among others, the following comorbid conditions: stroke, heart attack, angina pectoris/coronary heart disease, heart failure, arthrosis, rheumatism, osteoporosis, depression, and new primary cancer. The same comorbid conditions were assessed in the LinDe study, except for new primary cancer.

### Sociodemographic and clinical data

Participants self-reported their demographic characteristics (sex, age at survey, education, partnership status), lifestyle factors (smoking status, alcohol consumption, body mass index (BMI), and physical activity), and chronic health conditions at the time of the survey in both CAESAR and LinDe questionnaires. Socioeconomic deprivation was estimated by linking the 2022 version of the German Index of Socioeconomic Deprivation (GISD) to the Official Municipality Code (Amtliche Gemeindekennzahl, GKZ) [35, 36]. The cancer registries provided detailed information on cancer diagnosis, including cancer type, year of diagnosis, and stage at diagnosis of the CAESAR participants. Treatment and recurrence/metastasis information were obtained through self-reports and, for a subset of survivors, from their physicians.

### Statistical analyses

Descriptive analyses were conducted to summarise the demographic and clinical characteristics of participants, both in crude form and after age adjustment using direct standardisation with specific proportions from the LTCS sample serving as standard population weights. Differences between LTCS and cancer-free controls were assessed using Cochran-Mantel-Haenszel (CMH) tests and Student’s t-tests, where appropriate.

In the analyses involving only the LTCS from the CAESAR cohort, multiple linear regression was performed to evaluate whether comorbid DM (prevalent at baseline) was differentially associated with HRQOL at baseline (cross-sectional association) and follow-up (prospective association). Linear mixed regression models were employed with an interaction term (“DM” x “round of HRQOL assessment”) to examine whether changes in HRQOL over time could be attributed to the presence of DM at baseline in LTCS.

In a separate cross-sectional analysis based on a joint sample including both cancer survivors at baseline and cancer-free controls, linear mixed regression models were used to evaluate if the differences in mean HRQOL scores between those with cancer and those without were greater, similar, or smaller than the differences between diabetic and non-diabetic individuals. We also incorporated an interaction term (“DM” x “Cancer”) to investigate whether the cross-sectional association between DM and HRQOL differed between cancer survivors and cancer-free controls. Subgroup analyses by age groups at baseline and sex were conducted for summary scores, global health status, and financial difficulties.

Covariates selected a priori for adjustment in models with only CAESAR participants included: age at survey, sex, education, partnership, socioeconomic deprivation, BMI, physical activity, and comorbidity status (number of comorbidities out of those listed above, excluding DM). In addition, covariates such as alcohol consumption, smoking status, age at cancer diagnosis, cancer stage, and recurrence/metastasis were included in analysis with CAESAR participants only, as these variables were unavailable in LinDe. Tumour and sex were combined to one adjustment variable (breast female, colorectal female, colorectal male, prostate male) to avoid empty cells in the models.

Sensitivity analyses were performed to evaluate whether the cross-sectional association between DM prevalence at baseline and HRQOL at baseline varied according to participation at follow-up to account for potential effect modification due to differences in participation and survival. We also conducted sensitivity analyses on the association between DM and HRQOL at follow-up according to the time point of DM diagnosis (prevalent at baseline or incident during follow-up. Survivors who reported no DM at baseline but indicated DM at follow-up were classified as incident cases of DM during the study period.

A *p* < 0.05 (two-sided) was considered statistically significant. All analyses were carried out with SAS version 9.4 for Windows^®^ (SAS Institute Inc., Cary, NC, USA).

## Results

### Characteristics of participants in CAESAR and LinDe

A total of 6811 LTCS from CAESAR baseline (2008–4rr2011) were included in our analyses. Of these, 4,201 were alive and were invited to participate in the follow-up study (2018/2019). Among them, 2,699 (64% response) returned a full follow-up questionnaire, and of whom 2,27 who provided information on their DM status were included in the longitudinal analyses. Additionally, 1701 cancer-free individuals were included as population controls.

For LTCS, the mean age ( ± SD) was 69.0 ± 8.9 years, and the mean time since cancer diagnosis ( ± SD) was 8.0 ± 2.2 years at baseline. At baseline, 13.7% of LTCS indicated they had DM. LTCS with DM at baseline were older than LTCS without DM (table S1) and were less likely to participate in the follow-up round than those without DM (OR = 0.74, 95% CI 0.61–0.91) (data not shown). Among cancer-free controls, the mean age at survey was 60.1 ± 14.8 years, and of whom 10.5% indicated they had DM (Table S2).

After age standardisation, a higher proportion of participants were female among the LTCS group. Additionally, LTCS were more likely to have lower educational level, reside in regions with moderate socioeconomic deprivation, less likely to have a partner, and they reported more comorbidities than cancer-free controls. LTCS reported higher levels of and less missing data for physical activity. The distribution of BMI was similar among LTCS and cancer-free controls (Table 1).

**Table 1:** Characteristics of participants in CAESAR (long-term cancer survivors) and LinDe (cancer-free controls).

	CAESAR (*n* = 6811)		LinDe (*n* = 1701)			*p* value (CAESAR vs. LinDe)	
	*n*	%_crude_	*n*	%_crude_	%_adj_^a^	Crude	Adjusted^b^
Age at survey (years)						**<0.001**	-
<60	1028	15.1	821	48.3	-		
60–69	2043	30.0	359	21.1	-		
70–79	3087	45.3	303	17.8	-		
≥80	653	9.6	218	12.8	-		
Mean ± SD	69.0 ± 8.9		60.1 ± 14.8			**<0.001**	-
Sex						0.4	**<0.001**
Male	3219	47.3	783	46.0	51.1		
Female	3592	52.7	918	54.0	48.9		
Education (years)						**<0.001**	**<0.001**
≤9	3696	54.3	586	34.5	44.5		
10–11	1528	22.4	478	28.1	22.3		
≥12	1462	21.5	601	35.3	30.2		
Missing	125	1.8	36	2.1	2.9		
Socioeconomic deprivation^c^						**<0.001**	**<0.001**
1 (low)	454	6.7	418	24.6	25.0		
2	1999	29.4	329	19.3	18.0		
3	1976	29.0	255	15.0	15.4		
4	1879	27.6	372	21.9	21.7		
5 (high)	503	7.4	302	17.8	18.6		
In partnered relationship						**<0.001**	**<0.001**
No	1188	17.4	358	21.1	22.3		
Yes	5319	78.1	1314	77.3	75.6		
Missing	304	4.5	29	1.7	2.1		
BMI (kg/m^2^)						**<0.001**	0.2
<25	2421	35.6	704	41.4	34.9		
25- < 30	2917	42.8	644	37.9	43.4		
≥30	1266	18.6	319	18.8	19.9		
Missing	207	3.0	34	2.0	1.9		
Physical activity^d^						**<0.001**	**<0.001**
Insufficient	805	11.8	528	31.0	29.7		
Sufficient	4753	69.8	1110	65.3	66.9		
Missing	1253	18.4	63	3.7	3.5		
Comorbidity status^e^						**<0.001 **^**f**^	**0.009 **^**f**^
0	3113	45.7	927	54.5	45.3		
1	2095	30.8	460	27.0	29.8		
≥2	1603	23.5	314	18.5	24.8		

*CAESAR* Cancer Survivorship–A Multi-regional Population-Based Study, *LinDe* Lebensqualität in Deutschland (Quality of Life in Germany), *BMI* body mass index, *GISD* German index of socioeconomic deprivation.

^a^ Using the age specific proportions from the CAESAR sample as standard population weights.

^b^ Adjusted for age by Cochran-Mantel-Haenszel test.

^**c**^ Socioeconomic deprivation was based on the quintiles of the overall GISD. Upper quintiles indicate higher socioeconomic deprivation. 25 GISD values were missing for LinDe cohort.

^d^ Physical activity: Insufficient (moderate plus vigorous intensity <150 min/week), sufficient (moderate plus vigorous intensity ≥150 min/week).

^e^ The number of other comorbidities including stroke, heart attack, angina pectoris/coronary heart disease, heart failure, arthrosis, rheumatism, osteoporosis, depression.

^f^ The significant *p*-value indicates variations in comorbidity distribution within specific age groups, despite similar overall percentages between cancer survivors and cancer-free participants. LinDe is not a random sample of the general population. An age-stratified sampling scheme was employed to obtain sufficiently large samples for age-specific analyses.

**Bold** ***p*-values** show statistically significant differences (*p* < 0.05).

### The association between prevalent DM at baseline and HRQOL at baseline and follow-up

**Fig. 2:**
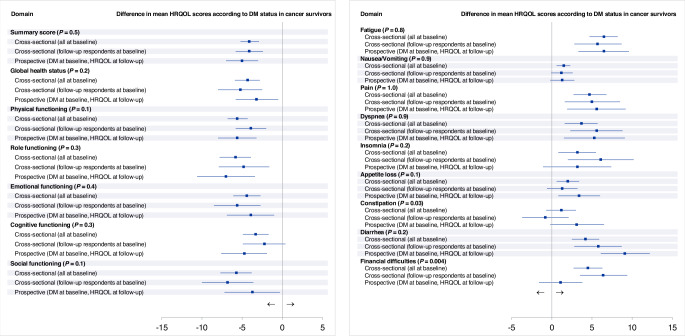
Difference in mean health-related quality of life (HRQOL) scores in cancer survivors at baseline (cross-sectional) and at follow-up (prospective) according to diabetes mellitus (DM) status (yes versus no) at baseline. Footnote: Differences in mean HRQOL scores and the 95% confidence intervals are displayed as forest plots (non-DM group serving as the reference group). The displayed *p*-values reflect the interaction between DM and the study round (baseline and follow-up). All results were adjusted for age at survey, age at cancer diagnosis, tumour-sex, education, socioeconomic deprivation, partnership, body mass index, alcohol consumption, smoking status, physical activity, comorbidity status (the number of other comorbidities excluding DM), cancer stage, and recurrence/metastasis.

At baseline, LTCS with DM consistently reported poorer HRQOL with lower scores across all functioning scales, as well as on the summary score and global health status, and higher symptom scores compared to those without DM (Fig. 2). For the subgroup of survivors who participated in both rounds (*n* = 2627, denoted as “follow-up respondents at baseline”), the pattern of the association at baseline was similar to that of the total population.

The prospective association between DM status at baseline and HRQOL at subsequent follow-up was similar to the cross-sectional association measured at baseline, except for constipation and financial difficulties (Fig. 2). We observed a change in the direction of the association between DM at baseline and constipation when comparing cross-sectional and prospective findings (p_interaction_ = 0.03). Additionally, the prospective association of DM at baseline with financial difficulties weakened over time (p_interaction_ = 0.004) compared to the baseline cross-sectional findings (Fig. 2).

### Comparing the association between DM and HRQOL in LTCS and cancer-free controls

Both cancer and DM were associated with poorer HRQOL in cross-sectional analyses at baseline in the joint sample of LTCS and cancer-free controls (Table 2, Fig. S1). The association was independent between cancer and DM for most scales except for emotional functioning. Cancer survivors with DM reported worse emotional functioning than expected from the individual associations observed for cancer and DM alone (*p* = 0.046).

**Table 2:** Differences in mean HRQOL scores according to cancer, DM status, and cancer*DM, involving both cancer survivors at baseline and cancer-free controls (cross-sectional at baseline).

	Cancer			DM			Cancer * DM ^a^		
	Estimate	StdErr	*P* value	Estimate	StdErr	*P* value	Estimate	StdErr	*P* value
Physical functioning	−4.7	0.6	< 0.001	−6.3	1.4	< 0.001	0.4	1.5	0.8
Role functioning	−6.8	0.9	< 0.001	−4.9	2.2	0.02	−1.1	2.4	0.6
Emotional functioning	−3.4	0.8	< 0.001	−0.7	1.9	0.7	−4.1	2.1	0.046
Cognitive functioning	−4.3	0.7	< 0.001	−1.5	1.8	0.4	−1.6	1.9	0.4
Social functioning	−9.7	0.9	< 0.001	−9.2	2.1	< 0.001	3.3	2.3	0.2
Global health status	−3.2	0.7	< 0.001	−6.9	1.7	< 0.001	2.5	1.9	0.2
Fatigue	4.9	0.8	< 0.001	3.8	1.9	0.1	3.2	2.1	0.1
Nausea/Vomiting	1.2	0.4	0.02	0.5	0.9	0.6	1.2	1.0	0.3
Pain	1.7	0.9	0.1	7.2	2.3	0.002	−2.4	2.4	0.3
Dyspnea	7.5	0.9	< 0.001	3.0	2.2	0.2	1.5	2.4	0.5
Insomnia	6.2	1.1	< 0.001	1.0	2.6	0.7	2.3	2.8	0.4
Appetite loss	2.8	0.6	< 0.001	4.6	1.5	0.003	−2.4	1.7	0.2
Constipation	5.5	0.8	< 0.001	2.5	2.0	0.2	−0.9	2.1	0.7
Diarrhea	6.8	0.8	< 0.001	3.3	1.8	0.1	1.2	2.0	0.6
Financial difficulties	6.1	0.8	< 0.001	6.5	2.0	0.001	−1.9	2.2	0.4
Summary score	−4.9	0.5	< 0.001	−3.7	1.2	0.002	−0.6	1.3	0.7

All results were adjusted for age, sex, education, socioeconomic deprivation, partnership, body mass index, physical activity, and comorbidity status (the number of other comorbidities excluding DM and the second primary tumour) at survey.

*DM* diabetes mellitus, *HRQOL* health-related quality of life, *StdErr* standard error.

^a^Reference group: non-cancer, non-DM.

The association between cancer and HRQOL was stronger than or similar to the association between DM and HRQOL across most domains. However, DM appeared to exhibit a stronger association with HRQOL than cancer in specific domains such as physical functioning, global health status, pain, and appetite loss (Table 2). However, caution is warranted when interpreting these findings due to larger standard errors observed in the calculation of differences in mean HRQOL scores by DM status compared to cancer status across all domains (as well as in the subsequent subgroup analyses, Figs. S2 and S3).

Since the HRQOL summary score was derived from the arithmetic mean of 13 scales, excluding the global health/QoL and financial difficulties scales, the subsequent subgroup analyses included only the summary score, global health status, and financial difficulties. In stratified analyses, the patterns of association between cancer or DM with HRQOL might vary based on sociodemographic factors such as age and sex, particularly among participants below 80 years of age (Table S3).

Results of the cross-sectional analyses between DM and HRQOL at follow-up are shown in Tables S4 and S5, and Fig. S4.

## Discussion

In this study, we analysed the association of DM and cancer with HRQOL in a population-based sample of LTCS up to 24 years post-diagnosis, alongside a cancer-free comparison group. Given the limited evidence in this topic using population-based data with extended follow-up, our findings provide valuable insights. We found that both cancer and DM were associated with poorer HRQOL during long-term cancer survivorship. While the association between cancer and HRQOL was generally stronger than or comparable to that between DM and HRQOL across most domains, DM showed a stronger association with HRQOL in specific areas such as physical functioning, global health status, pain, and appetite loss. Additionally, as time since cancer diagnosis increased, the association between cancer and poorer HRQOL appeared to diminish.

It is well known that DM patients typically experience worse HRQOL compared to non-diabetics, particularly in physical domains [37]. This association between DM and HRQOL was also evident in our cohort of LTCS. Additionally, our study revealed impairments in HRQOL across mental and social health domains, which might be attributed to the effects of glucose metabolism disorders on brain function [38], the comorbidities of DM, and symptoms arising from the side effects of DM treatments.

For example, gastrointestinal dysfunction, particularly diarrhea, is a prevalent condition among persons with DM [39]. The underlying causes include DM-related autonomic neuropathy and side effects from medications such as metformin. Increased instances of diarrhea in LTCS with DM could adversely affect social functioning. One previous research has shown a significant, although modest, negative correlation between diarrhea and social functioning [40]. This indicated that the fear and embarrassment related to potential issues like stool leakage and faecal odour could impact partnership, sexuality, and social interactions, and potentially hinder survivors from seeking help [41, 42]. Additionally, LTCS with DM exhibited more sleep problems, which may be linked to the broader mental challenges [43]. The interplay of mental and physical health issues also contributes to elevated fatigue levels [44, 45].

While cancer is widely recognised as a life-threatening disease with significant associations with poorer HRQOL, our study demonstrates that, within 5-14 years post-diagnosis, DM can exhibit associations with HRQOL that are comparable to or even more pronounced than those of cancer. Specifically, DM is linked more strongly with declines in physical functioning, global health status, pain, and appetite loss. In a prior study involving colorectal cancer survivors from the same cohort as our study, it was reported that LTCS exhibited lower levels of pain when compared with population controls [40]. Extending this research, our current analysis revealed a stronger association of pain with DM than cancer. Regarding appetite loss, although cancer treatments such as chemotherapy and the progression of anorexia-cachexia syndrome are known contributors to this symptom [46], our observations suggest that in the long-term, DM plays a more significant role in ongoing appetite issues, possibly due to DM complications and the use of medications such as metformin [39, 47], Further, these symptoms can significantly impair physical functioning and global health status.

One previous study indicated that colorectal cancer survivors experience a more significant decrease in HRQOL compared to those affected by DM, with exceptions in physical activity, global health status, and pain [28]. This result is similar to our study. However, our longitudinal study, which tracked LTCS for up to 24 years post-diagnosis, offers more detailed insights. It showed that while the association of DM with HRQOL remained relatively stable over time, the association of cancer with poorer HRQOL tended to diminish. Initially, cancer was associated with a roughly 5-point lower mean summary score compared to 3.7 points for DM. At follow-up, this score was down-adjusted to 2.7 for cancer, whereas it remained at 3.7 for DM. Similarly, increased financial difficulties related to both conditions were comparable at baseline; however, by follow-up, these issues became more pronounced for DM (a 1.7-point increase for cancer compared to a 6.0-point increase for DM) (Table S4).

This observation underscores that LTCS may experience significant challenges not only from cancer but also from comorbid conditions such as DM. This could lead to increased healthcare utilisation, as comorbidities like DM are associated with higher utilisation of healthcare in LTCS over extended periods post-cancer diagnosis [48]. It emphasises the need to improve the management of these concurrent health conditions and to reduce the side effects of their treatment.

However, our findings comparing the associations of cancer and DM with HRQOL need a cautious interpretation. During the follow-up period, 121 new cases of DM were documented, which likely contributed to the stronger association observed with HRQOL at follow-up for DM. A sensitivity analysis indicated that the associations of both newly diagnosed and previously existing DM with HRQOL at follow-up were similar (Fig. S3). These findings imply that the long-term associations of cancer with HRQOL decrease more rapidly than those of DM with HRQOL, potentially due to insufficient management of comorbid conditions in long-term survivors [49]. In addition, the inclusion of the same non-cancer control group in both baseline and follow-up analyses might have obscured the evolving nature of the DM-HRQOL association over time. Nonetheless, no meaningful significant interaction between cancer and DM concerning HRQOL was detected, indicating that the relationship between DM and HRQOL does not significantly differ between LTCS and cancer-free individuals (Table 2 and Table S4). Furthermore, the larger standard errors observed in the associations between DM and HRQOL, compared to those between cancer and HRQOL, indicate greater variability in the DM-HRQOL associations. This variability necessitates caution in drawing conclusions from our results and underscores the complexity of managing DM with or without cancer. It also emphasises the importance of developing a standardised management strategy for all cancer survivors with DM to address these varied outcomes effectively. Previous studies suggest that adopting a healthier lifestyle could improve HRQOL, irrespective whether the individual has cancer, DM, or both diseases [50, 51]. Of note, it should be highlighted to cancer survivors with DM not to neglect the management of one condition over the other during cancer survivorship [52].

Our study has several limitations. The data regarding DM were primarily based on self-report, but our previous research indicated high concordance between patient-reported and physician-reported DM in LTCS [53], supporting the validity of self-reported DM. Secondly, there may be an underestimation of the HRQOL in LTCS with DM at follow-up due to selective survival. In our previous publication, we observed a higher mortality rate among LTCS with DM than those without DM during a median follow-up time of 10.2 years [53]. Furthermore, despite adjusting the regression models for a broad range of covariates, there remains the potential for residual confounding, e.g., we do not have information on lifestyle behaviours in the control group nor indicators of diabetes self-management. Additionally, the EORTC QLQ-C30 may not comprehensively capture all dimensions of HRQOL relevant for LTCS. To address this, future research could incorporate the EORTC QLQ-Cancer Survivorship questionnaire, offering a more nuanced evaluation of HRQOL among LTCS [54]. Moreover, this study does not distinguish between Type 1 Diabetes Mellitus (T1DM) and Type 2 Diabetes Mellitus (T2DM). Since T2DM is prevalent, especially among the older age groups included in this study, and the association of T1DM and T2DM with HRQOL may be similar [55], consequently, our findings may be reflective of the association between T2DM and HRQOL in LTCS. In addition, the duration and severity of DM should be carefully considered in future research.

In summary, LTCS with DM generally reported lower levels of HRQOL compared to their non-diabetic counterparts. For some domains, this association was observed to be more pronounced than the association between cancer and HRQOL. This underscores the necessity for comprehensive care strategies that incorporate management of both cancer and concurrent chronic conditions like DM to improve overall health outcomes for LTCS.

## Supplementary information

Supplementary Tables and Figures

Reproducibility checklist

## Data Availability

Data is available from the authors upon reasonable request.
