# Supplementary material for: Diabetes mellitus and health-related quality of life in long-term survivors of breast, colorectal or prostate cancer: a population-based prospective study
Source: Br J Cancer. 2026 May 13;135(4):641–8. doi: 10.1038/s41416-026-03463-5 (PMC13427720; doi:10.1038/s41416-026-03463-5)
Supplement: Supplementary file 1 — Supplementary Tables and Figures [file 41416_2026_3463_MOESM1_ESM.docx]

Table S1. Characteristics of CAESAR participants according to DM status

|  | **Overall**  **(n = 6,811)** | | **Baseline without DM**  **(n = 5,881)** | | | **Baseline with DM**  **(n = 930)** | | | **P value** | |
| --- | --- | --- | --- | --- | --- | --- | --- | --- | --- | --- |
|  | **n** | **%** | **n** | **%_crude_** | **%_adjusted_^#^** | **n** | **%_crude_** | **%_adjusted_^#^** | **crude** | **adjusted^#^** |
| **Cancer site** |  |  |  |  |  |  |  |  | **<0.001** | **0.01** |
| Breast | 2,977 | 43.7 | 2,666 | 45.3 | 44.2 | 311 | 33.4 | 37.3 |  |  |
| Colorectal | 1,475 | 21.7 | 1,235 | 21.0 | 21.2 | 240 | 25.8 | 26.9 |  |  |
| Prostate | 2,359 | 34.6 | 1,980 | 33.7 | 34.6 | 379 | 40.8 | 35.8 |  |  |
| **Sex** |  |  |  |  |  |  |  |  | **<0.001** | **0.01** |
| Male | 3,219 | 47.3 | 2,683 | 45.6 | 46.7 | 536 | 57.6 | 53.7 |  |  |
| Female | 3,592 | 52.7 | 3,198 | 54.4 | 53.3 | 394 | 42.4 | 46.3 |  |  |
| **Age at survey (years)** | |  |  | |  |  | |  | **<0.001** | - |
| <60 | 1,028 | 15.1 | 981 | 16.7 | - | 47 | 5.1 | - |  |  |
| 60-69 | 2,043 | 30.0 | 1,822 | 31.0 | - | 221 | 23.8 | - |  |  |
| 70-79 | 3,087 | 45.3 | 2,543 | 43.2 | - | 544 | 58.5 | - |  |  |
| ≥80 | 653 | 9.6 | 535 | 9.1 | - | 118 | 12.7 | - |  |  |
| Mean ± SD | 69.0 ± 8.9 | | 68.5 ± 9.1 | | | 72.2 ± 6.8 | | | **<0.001** | - |
| **Time since cancer diagnosis (years)** | | |  | |  |  | |  | 0.3 | 0.4 |
| <10 | 5,328 | 78.2 | 4,604 | 78.3 | 78.2 | 724 | 77.9 | 79.3 |  |  |
| 10-16 | 1,442 | 21.2 | 1,245 | 21.2 | 21.3 | 197 | 21.2 | 19.9 |  |  |
| Missing | 41 | 0.6 | 32 | 0.6 | 0.6 | 9 | 1.0 | 0.8 |  |  |
| Mean ± SD | 8.0 ± 2.2 | | 8.0 ± 2.2 | | | 8.0 ± 2.2 | | | 0.5 | - |
| **Education (years)** |  |  |  | |  |  | |  | **<0.001** | **<0.001** |
| ≤9 | 3,696 | 54.3 | 3,086 | 52.5 | 52.9 | 610 | 65.6 | 65.6 |  |  |
| 10 - 11 | 1,528 | 22.4 | 1,366 | 23.2 | 23.0 | 162 | 17.4 | 17.1 |  |  |
| ≥12 | 1,462 | 21.5 | 1,325 | 22.5 | 22.4 | 137 | 14.7 | 15.3 |  |  |
| Missing | 125 | 1.8 | 104 | 1.8 | 1.8 | 21 | 2.3 | 1.9 |  |  |
| **Socioeconomic deprivation^&^** | |  |  |  |  |  |  |  | **0.04** | **0.01** |
| 1 (low) | 454 | 6.7 | 414 | 7.0 | 7.1 | 40 | 4.3 | 4.2 |  |  |
| 2 | 1,999 | 29.4 | 1,727 | 29.4 | 29.5 | 272 | 29.3 | 29.3 |  |  |
| 3 | 1,976 | 29.0 | 1,699 | 28.9 | 28.9 | 277 | 29.8 | 29.2 |  |  |
| 4 | 1,879 | 27.6 | 1,607 | 27.3 | 27.2 | 272 | 29.3 | 30.1 |  |  |
| 5 (high) | 503 | 7.4 | 434 | 7.4 | 7.4 | 69 | 7.4 | 7.3 |  |  |
| **In a partnered relationship** | |  |  |  |  |  |  |  | **<0.001** | **<0.001** |
| No | 1,188 | 17.4 | 1,023 | 17.4 | 17.6 | 165 | 17.4 | 16.2 |  |  |
| Yes | 5,319 | 78.1 | 4,619 | 78.6 | 78.3 | 700 | 75.3 | 76.4 |  |  |
| Missing | 304 | 4.5 | 239 | 4.1 | 4.1 | 65 | 7.0 | 7.4 |  |  |
| **BMI (kg/m^2^)** |  |  |  |  |  |  |  |  | **<0.001** | **<0.001** |
| <25 | 2,421 | 35.6 | 2,255 | 38.4 | 38.0 | 166 | 17.9 | 16.2 |  |  |
| 25-<30 | 2,917 | 42.8 | 2,509 | 42.7 | 42.9 | 408 | 43.9 | 43.0 |  |  |
| ≥30 | 1,266 | 18.6 | 946 | 16.1 | 16.1 | 320 | 34.4 | 37.3 |  |  |
| Missing | 207 | 3.0 | 171 | 2.9 | 3.0 | 36 | 3.9 | 3.5 |  |  |
| **Alcohol consumption** | |  |  |  |  |  |  |  | **<0.001** | **<0.001** |
| No | 2,210 | 32.5 | 1,886 | 32.1 | 32.2 | 324 | 34.8 | 34.8 |  |  |
| Former | 563 | 8.3 | 452 | 7.7 | 7.8 | 111 | 11.9 | 11.8 |  |  |
| Current | 3,075 | 45.2 | 2,763 | 47.0 | 46.7 | 312 | 33.6 | 32.7 |  |  |
| Missing | 963 | 14.1 | 780 | 13.3 | 13.3 | 183 | 19.7 | 20.6 |  |  |
| **Smoking status** |  |  |  |  |  |  |  |  | **<0.001** | **<0.001** |
| No | 4,667 | 68.5 | 4,070 | 69.2 | 69.7 | 597 | 64.2 | 60.6 |  |  |
| Former | 609 | 8.9 | 516 | 8.8 | 8.7 | 93 | 10.0 | 10.4 |  |  |
| Current | 613 | 9.0 | 548 | 9.3 | 9.0 | 65 | 7.0 | 9.1 |  |  |
| Missing | 922 | 13.5 | 747 | 12.7 | 12.7 | 175 | 18.8 | 19.9 |  |  |
| **Physical activity^*^** |  |  |  |  |  |  |  |  | **<0.001** | **<0.001** |
| Insufficient | 805 | 11.8 | 644 | 11.0 | 11.2 | 161 | 17.3 | 14.7 |  |  |
| Sufficient | 4,753 | 69.8 | 4,228 | 71.9 | 71.5 | 525 | 56.5 | 58.1 |  |  |
| Missing | 1,253 | 18.4 | 1,009 | 17.2 | 17.3 | 244 | 26.2 | 27.2 |  |  |
| **Comorbidity status^$^** | |  |  |  |  |  |  |  | **<0.001** | **<0.001** |
| 0 | 2,765 | 40.6 | 2,483 | 42.2 | 42.0 | 282 | 30.3 | 31.9 |  |  |
| 1 | 2,178 | 32.0 | 1,880 | 32.0 | 32.0 | 298 | 32.0 | 33.7 |  |  |
| ≥2 | 1,868 | 27.4 | 1,518 | 25.8 | 26.1 | 350 | 37.6 | 34.4 |  |  |
| **Recurrence/metastasis** | |  |  |  |  |  |  |  | **0.04** | 0.2 |
| No | 5,232 | 76.8 | 4,542 | 77.2 | 77.1 | 690 | 74.2 | 74.4 |  |  |
| Yes | 1,579 | 23.2 | 1,339 | 22.8 | 22.9 | 240 | 25.8 | 25.6 |  |  |
| **Cancer stage** |  |  |  |  |  |  |  |  | 0.2 | 0.6 |
| I / II | 4,234 | 62.2 | 3,674 | 62.5 | 62.0 | 560 | 60.2 | 61.8 |  |  |
| III / IV | 1,306 | 19.2 | 1,131 | 19.2 | 19.4 | 175 | 18.8 | 19.6 |  |  |
| Unknown | 1,271 | 18.7 | 1,076 | 18.3 | 18.6 | 195 | 21.0 | 18.5 |  |  |
| **Abbreviations:** CAESAR, Cancer Survivorship - A Multi-regional Population-Based Study; BMI, body mass index; DM, diabetes mellitus; GISD, German index of socioeconomic deprivation.  ^&^ Socioeconomic deprivation was based on the quintiles of the overall GISD, upper quintiles indicate higher socioeconomic deprivation.  ^*^ Physical activity: Insufficient (moderate plus vigorous intensity <150 min/week), sufficient (moderate plus vigorous intensity ≥150 min/week).  **^$^** The number of other comorbidities except DM.  ^#^ Adjusted for age. | | | | | | | | | | |

Table S2. Characteristics of LinDe participants according to DM status

|  | **Overall**  **(n = 1,701)** | | **Baseline without DM**  **(n = 1,522)** | | | **Baseline with DM**  **(n = 179)** | | | **P value** | |
| --- | --- | --- | --- | --- | --- | --- | --- | --- | --- | --- |
|  | **n** | **%** | **n** | **%_crude_** | **%_adjusted_^#^** | **n** | **%_crude_** | **%_adjusted_^#^** | **crude** | **adjusted^#^** |
| **Sex** |  |  |  |  |  |  |  |  | **<0.001** | **0.03** |
| Male | 783 | 46.0 | 678 | 44.6 | 45.1 | 105 | 58.7 | 52.1 |  |  |
| Female | 918 | 54.0 | 844 | 55.5 | 54.9 | 74 | 41.3 | 47.9 |  |  |
| **Age at survey (years)** | |  |  | |  |  | |  | **<0.001** | - |
| <60 | 821 | 48.3 | 786 | 51.6 | - | 35 | 19.6 | - |  |  |
| 60-69 | 359 | 21.1 | 311 | 20.4 | - | 48 | 26.8 | - |  |  |
| 70-79 | 303 | 17.8 | 250 | 16.4 | - | 53 | 29.6 | - |  |  |
| ≥80 | 218 | 12.8 | 175 | 11.5 | - | 43 | 24.0 | - |  |  |
| Mean ± SD | 60.1 ± 14.8 | | 59.0 ± 14.8 | | | 69.0 ± 11.9 | | | **<0.001** | - |
| **Education (years)** |  |  |  | |  |  | |  | **<0.001** | 0.2 |
| ≤9 | 586 | 34.5 | 495 | 32.5 | 33.6 | 91 | 50.8 | 46.1 |  |  |
| 10 - 11 | 478 | 28.1 | 441 | 29.0 | 28.4 | 37 | 20.7 | 27.2 |  |  |
| ≥12 | 601 | 35.3 | 555 | 36.5 | 35.8 | 46 | 25.7 | 23.8 |  |  |
| Missing | 36 | 2.1 | 31 | 2.0 | 2.1 | 5 | 2.8 | 3.0 |  |  |
| **Socioeconomic deprivation^&^** | |  |  |  |  |  |  |  | 0.6 | 0.6 |
| 1 (low) | 418 | 24.6 | 380 | 25.0 | 25.0 | 38 | 21.2 | 19.4 |  |  |
| 2 | 329 | 19.3 | 294 | 19.3 | 19.1 | 35 | 19.6 | 16.8 |  |  |
| 3 | 255 | 15.0 | 231 | 15.2 | 15.3 | 24 | 13.4 | 14.7 |  |  |
| 4 | 372 | 21.9 | 332 | 21.8 | 21.9 | 40 | 22.4 | 27.9 |  |  |
| 5 (high) | 302 | 17.8 | 262 | 17.2 | 17.3 | 40 | 22.4 | 20.3 |  |  |
| Missing | 25 | 1.5 | 23 | 1.5 | 1.5 | 2 | 1.1 | 0.9 |  |  |
| **In a partnered relationship** | |  |  |  |  |  |  |  | **<0.001** | 0.08 |
| No | 358 | 21.1 | 304 | 20.0 | 20.4 | 54 | 30.2 | 34.5 |  |  |
| Yes | 1,314 | 77.3 | 1,195 | 78.5 | 78.0 | 103 | 66.5 | 63.6 |  |  |
| Missing | 29 | 1.7 | 23 | 1.5 | 1.6 | 11 | 3.4 | 1.9 |  |  |
| **BMI (kg/m^2^)** |  |  |  |  |  |  |  |  | **<0.001** | **<0.001** |
| <25 | 704 | 41.4 | 681 | 44.7 | 44.3 | 23 | 12.9 | 11.9 |  |  |
| 25-<30 | 644 | 37.9 | 570 | 37.5 | 37.9 | 74 | 41.3 | 35.6 |  |  |
| ≥30 | 319 | 18.8 | 241 | 15.8 | 15.9 | 78 | 43.6 | 51.3 |  |  |
| Missing | 34 | 2.0 | 30 | 2.0 | 2.0 | 4 | 2.2 | 1.3 |  |  |
| **Physical activity^*^** |  |  |  |  |  |  |  |  | **0.03** | 0.1 |
| Insufficient | 528 | 31.0 | 463 | 30.4 | 30.5 | 65 | 36.3 | 39.2 |  |  |
| Sufficient | 1,110 | 65.3 | 1,007 | 66.2 | 66.0 | 103 | 57.5 | 56.0 |  |  |
| Missing | 63 | 3.7 | 52 | 3.4 | 3.5 | 11 | 6.2 | 4.8 |  |  |
| **Comorbidity status^$^** | |  |  |  |  |  |  |  | **<0.001** | **<0.001** |
| 0 | 927 | 54.5 | 864 | 56.8 | 55.7 | 63 | 35.2 | 38.3 |  |  |
| 1 | 460 | 27.0 | 417 | 27.4 | 27.8 | 43 | 24.0 | 23.3 |  |  |
| ≥2 | 314 | 18.5 | 241 | 15.8 | 16.5 | 73 | 40.8 | 38.4 |  |  |
| **Abbreviations:** LinDe, Lebensqualität in Deutschland (Quality of Life in Germany); BMI, body mass index; DM, diabetes mellitus; GISD, German index of socioeconomic deprivation.  ^&^ Socioeconomic deprivation was based on the quintiles of the overall GISD, upper quintiles indicate higher socioeconomic deprivation.  ^*^ Physical activity: Insufficient (moderate plus vigorous intensity <150 min/week), sufficient (moderate plus vigorous intensity ≥150 min/week).  **^$^** The number of other comorbidities except DM.  ^#^ Adjusted for age. | | | | | | | | | | |

Table S3. Differences in mean HRQOL scores according to cancer, DM status, and cancer*DM, involving both cancer survivors at baseline and cancer-free controls, subgroup analyses (cross-sectional at baseline)

|  | Cancer | | | DM | | | Cancer * DM ^c^ | | |
| --- | --- | --- | --- | --- | --- | --- | --- | --- | --- |
|  | Estimate | StdErr | P value | Estimate | StdErr | P value | Estimate | StdErr | P value |
| **Summary score** |  |  |  |  |  |  |  |  |  |
| **By age ^a^** |  |  |  |  |  |  |  |  |  |
| <60 years | -5.5 | 0.8 | < 0.001 | -3.2 | 2.6 | 0.2 | -3.6 | 3.3 | 0.3 |
| 60 - <70 years | -3.9 | 0.9 | < 0.001 | -2.5 | 2.2 | 0.3 | -2.1 | 2.4 | 0.4 |
| 70 - <80 years | -4.1 | 1.1 | < 0.001 | -2.9 | 2.3 | 0.2 | -2.0 | 2.4 | 0.4 |
| ≥ 80 years | -3.1 | 1.6 | 0.1 | -3.8 | 3.0 | 0.2 | 2.3 | 3.5 | 0.5 |
| **By sex ^b^** |  |  |  |  |  |  |  |  |  |
| Male | -4.5 | 0.8 | < 0.001 | -4.3 | 1.6 | 0.01 | -0.9 | 1.7 | 0.6 |
| Female | -5.0 | 0.7 | < 0.001 | -2.5 | 1.9 | 0.2 | -0.6 | 2.1 | 0.8 |
|  |  |  |  |  |  |  |  |  |  |
| **Global health status** | |  |  |  |  |  |  |  |  |
| **By age ^a^** |  |  |  |  |  |  |  |  |  |
| <60 years | -2.1 | 1.2 | 0.1 | -7.1 | 3.8 | 0.1 | 2.9 | 4.9 | 0.6 |
| 60 - <70 years | -5.1 | 1.4 | < 0.001 | -6.5 | 3.3 | 0.1 | 0.3 | 3.6 | 0.9 |
| 70 - <80 years | -3.1 | 1.5 | 0.04 | -4.5 | 3.3 | 0.2 | -0.5 | 3.4 | 0.9 |
| ≥ 80 years | 0.2 | 2.0 | 0.9 | -7.8 | 3.7 | 0.04 | 7.6 | 4.3 | 0.1 |
| **By sex ^b^** |  |  |  |  |  |  |  |  |  |
| Male | -3.8 | 1.1 | < 0.001 | -7.5 | 2.3 | 0.001 | 2.4 | 2.5 | 0.3 |
| Female | -2.7 | 1.0 | 0.01 | -6.0 | 2.6 | 0.02 | 2.8 | 2.9 | 0.3 |
|  |  |  |  |  |  |  |  |  |  |
| **Financial difficulties** | |  |  |  |  |  |  |  |  |
| **By age ^a^** |  |  |  |  |  |  |  |  |  |
| <60 years | 11.0 | 1.5 | < 0.001 | 9.6 | 4.7 | 0.04 | -2.1 | 6.2 | 0.7 |
| 60 - <70 years | 2.7 | 1.6 | 0.1 | 8.2 | 3.8 | 0.03 | 2.2 | 4.2 | 0.6 |
| 70 - <80 years | 1.3 | 1.6 | 0.4 | 4.2 | 3.6 | 0.2 | -0.2 | 3.8 | 1.0 |
| ≥ 80 years | -0.4 | 2.1 | 0.9 | -7.8 | 4.0 | 0.1 | 6.3 | 4.6 | 0.2 |
| **By sex ^b^** |  |  |  |  |  |  |  |  |  |
| Male | 3.5 | 1.3 | 0.01 | 4.7 | 2.6 | 0.1 | -0.2 | 2.8 | 0.9 |
| Female | 7.2 | 1.1 | < 0.001 | 7.2 | 3.1 | 0.02 | -2.2 | 3.4 | 0.5 |
| Abbreviations: HRQOL, health-related quality of life; DM, diabetes mellitus; StdErr, standard error.  Footnotes:  ^a^ All results were adjusted for sex, education, socioeconomic deprivation, partnership, body mass index, physical activity and comorbidity status (the number of other comorbidities excluding DM and the second primary tumor) at survey.  ^b^ All results were adjusted for age, education, socioeconomic deprivation, partnership, body mass index, physical activity and comorbidity status (the number of other comorbidities excluding DM and the second primary tumor) at survey.  ^c^ Reference group: non-cancer, non-DM. | | | | | | | | | |

Table S4. Differences in mean HRQOL scores according to cancer, DM status, and cancer*DM, involving both cancer survivors at follow-up and non-cancer controls (cross-sectional at follow-up)

|  | Cancer | | | DM | | | Cancer * DM ^a^ | | |
| --- | --- | --- | --- | --- | --- | --- | --- | --- | --- |
|  | Estimate | StdErr | P value | Estimate | StdErr | P value | Estimate | StdErr | P value |
| Physical functioning | -1.7 | 0.7 | 0.02 | -5.5 | 1.4 | < 0.001 | -0.7 | 1.7 | 0.7 |
| Role functioning | -3.3 | 1.1 | 0.003 | -4.5 | 2.1 | 0.04 | -2.0 | 2.6 | 0.4 |
| Emotional functioning | -0.7 | 0.9 | 0.5 | -1.0 | 1.8 | 0.6 | -2.7 | 2.2 | 0.2 |
| Cognitive functioning | -1.9 | 0.9 | 0.03 | -1.0 | 1.7 | 0.6 | -3.7 | 2.1 | 0.1 |
| Social functioning | -5.8 | 1.1 | < 0.001 | -8.6 | 2.1 | < 0.001 | 4.8 | 2.5 | 0.1 |
| Global health status | 1.3 | 0.9 | 0.1 | -7.2 | 1.7 | < 0.001 | 3.7 | 2.0 | 0.1 |
| Fatigue | 3.1 | 1.0 | <0.001 | 4.3 | 1.9 | 0.02 | 2.3 | 2.3 | 0.3 |
| Nausea/Vomiting | 0.9 | 0.5 | 0.045 | 0.7 | 0.9 | 0.4 | 0.6 | 1.1 | 0.6 |
| Pain | -3.5 | 1.1 | 0.002 | 6.5 | 2.2 | 0.003 | -1.4 | 2.6 | 0.6 |
| Dyspnea | 4.6 | 1.1 | < 0.001 | 4.3 | 2.2 | 0.1 | 0.5 | 2.6 | 0.9 |
| Insomnia | 2.7 | 1.3 | 0.046 | 1.3 | 2.6 | 0.6 | 3.5 | 3.1 | 0.3 |
| Appetite loss | 1.7 | 0.8 | 0.04 | 4.7 | 1.6 | 0.003 | -1.5 | 1.9 | 0.4 |
| Constipation | 4.4 | 1.0 | < 0.001 | 2.8 | 1.9 | 0.1 | -1.0 | 2.3 | 0.7 |
| Diarrhea | 7.3 | 0.9 | < 0.001 | 3.6 | 1.7 | 0.03 | 4.6 | 2.1 | 0.03 |
| Financial difficulties | 1.7 | 0.9 | 0.1 | 6.0 | 1.7 | < 0.001 | -2.0 | 2.1 | 0.4 |
| Summary score | -2.7 | 0.6 | < 0.001 | -3.7 | 1.2 | 0.001 | -4.0 | 2.0 | 0.048 |
| Abbreviations: HRQOL, health-related quality of life; DM, diabetes mellitus; StdErr, standard error.  Footnote: All results were adjusted for age, sex, education, socioeconomic deprivation, partnership, body mass index, physical activity and comorbidity status (the number of other comorbidities excluding DM and the second primary tumor) at survey.  ^a^ Reference group: non-cancer, non-DM. | | | | | | | | | |

Table S5. Differences in mean HRQOL scores according to cancer, DM status, and cancer*DM, involving both cancer survivors at follow-up and non-cancer controls, subgroup analyses (cross-sectional at follow-up)

|  | Cancer | | | DM | | | Cancer * DM ^c^ | | |
| --- | --- | --- | --- | --- | --- | --- | --- | --- | --- |
|  | Estimate | StdErr | P value | Estimate | StdErr | P value | Estimate | StdErr | P value |
| **Summary score** |  |  |  |  |  |  |  |  |  |
| **By age ^a^** |  |  |  |  |  |  |  |  |  |
| <60 years | -4.8 | 1.2 | < 0.001 | -5.2 | 2.2 | 0.02 | -3.9 | 4.6 | 0.4 |
| 60 - <70 years | -3.1 | 1.1 | 0.01 | -1.6 | 2.2 | 0.5 | -3.5 | 3.1 | 0.3 |
| 70 - <80 years | -2.3 | 1.0 | 0.03 | -3.5 | 2.1 | 0.1 | -1.6 | 2.4 | 0.5 |
| ≥ 80 years | -0.1 | 1.4 | 1.0 | -3.3 | 2.8 | 0.3 | -1.2 | 3.2 | 0.7 |
| **By sex ^b^** |  |  |  |  |  |  |  |  |  |
| Male | -1.2 | 0.9 | 0.2 | -3.7 | 1.5 | 0.01 | -1.6 | 1.8 | 0.4 |
| Female | -3.5 | 0.8 | < 0.001 | -3.3 | 1.8 | 0.1 | -0.9 | 2.2 | 0.7 |
|  |  |  |  |  |  |  |  |  |  |
| **Global health status** | |  |  |  |  |  |  |  |  |
| **By age ^a^** |  |  |  |  |  |  |  |  |  |
| <60 years | 0.3 | 2.0 | 0.9 | -7.7 | 3.7 | 0.04 | 6.2 | 7.5 | 0.4 |
| 60 - <70 years | -0.2 | 1.7 | 0.9 | -5.5 | 3.2 | 0.1 | -2.9 | 4.7 | 0.5 |
| 70 - <80 years | 1.5 | 1.5 | 0.3 | -5.2 | 3.1 | 0.1 | -0.8 | 3.5 | 0.8 |
| ≥ 80 years | 5.0 | 1.9 | 0.01 | -7.4 | 3.6 | 0.04 | 6.4 | 4.0 | 0.1 |
| **By sex ^b^** |  |  |  |  |  |  |  |  |  |
| Male | 2.9 | 1.3 | 0.03 | -7.0 | 2.2 | 0.001 | 2.6 | 2.6 | 0.3 |
| Female | 0.5 | 1.2 | 0.7 | -6.3 | 2.6 | 0.01 | 4.1 | 3.1 | 0.2 |
|  |  |  |  |  |  |  |  |  |  |
| **Financial difficulties** | |  |  |  |  |  |  |  |  |
| **By age ^a^** |  |  |  |  |  |  |  |  |  |
| <60 years | 10.7 | 2.1 | < 0.001 | 10.9 | 3.8 | 0.004 | -12.5 | 7.8 | 0.1 |
| 60 - <70 years | 0.9 | 1.8 | 0.6 | 8.4 | 3.5 | 0.02 | 6.4 | 5.1 | 0.2 |
| 70 - <80 years | -2.2 | 1.4 | 0.1 | 5.2 | 2.9 | 0.1 | -4.4 | 3.3 | 0.2 |
| ≥ 80 years | -3.3 | 1.9 | 0.1 | -7.3 | 3.7 | 0.047 | 8.7 | 4.1 | 0.03 |
| **By sex ^b^** |  |  |  |  |  |  |  |  |  |
| Male | -1.5 | 1.3 | 0.3 | 4.5 | 2.2 | 0.04 | -4.1 | 2.6 | 0.1 |
| Female | 4.1 | 1.2 | < 0.001 | 7.0 | 2.7 | 0.01 | -2.5 | 3.2 | 0.4 |
| Abbreviations: HRQOL, health-related quality of life; DM, diabetes mellitus; StdErr, standard error.  Footnote:  ^a^ All results were adjusted for sex, education, socioeconomic deprivation, partnership, body mass index, physical activity and comorbidity status (the number of other comorbidities excluding DM and the second primary tumor) at survey.  ^b^ All results were adjusted for age, education, socioeconomic deprivation, partnership, body mass index, physical activity and comorbidity status (the number of other comorbidities excluding DM and the second primary tumor) at survey.  ^c^ Reference group: non-cancer, non-DM. | | | | | | | | | |


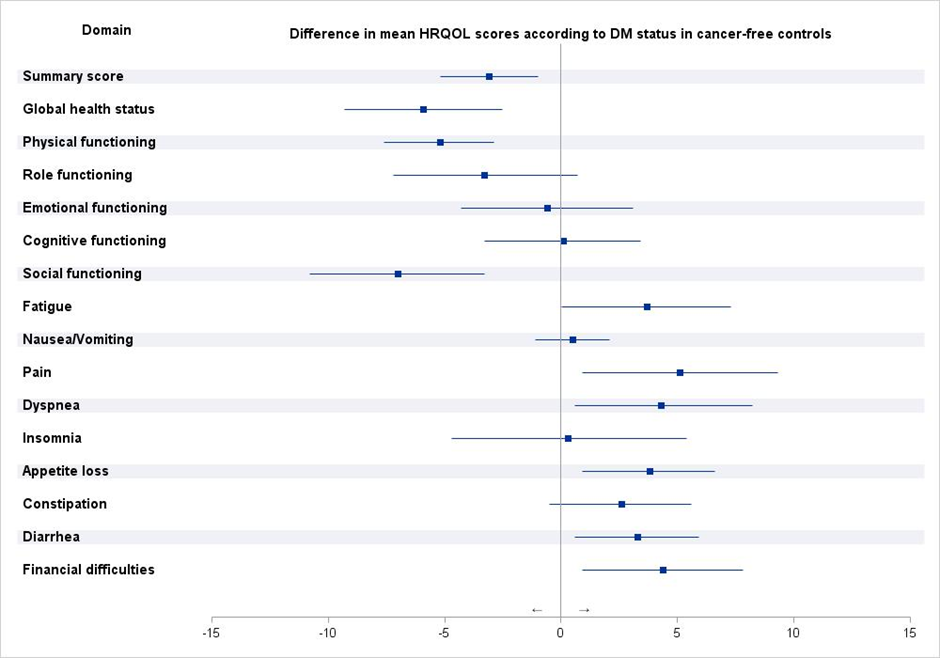


Figure S1. Difference in mean health-related quality of life (HRQOL) scores according to diabetes mellitus (DM) status in cancer-free controls

Footnote: difference in mean HRQOL scores and the 95% confidence intervals are displayed as forest plots (non-DM group served as the reference group). All results were adjusted for age at survey, sex, education, socioeconomic deprivation, partnership, body mass index, physical activity and comorbidity status (the number of other comorbidities except DM)


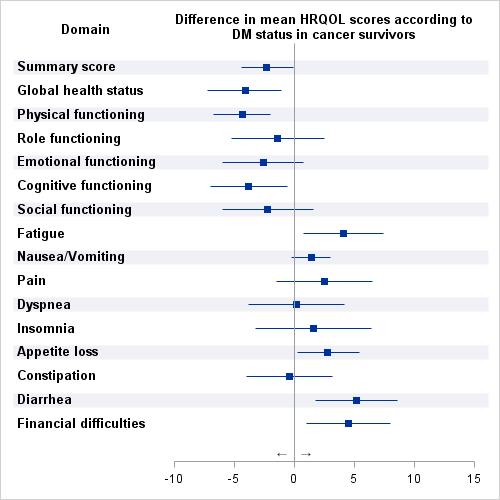

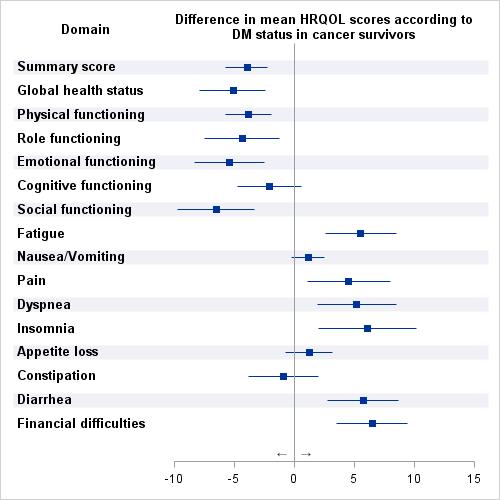

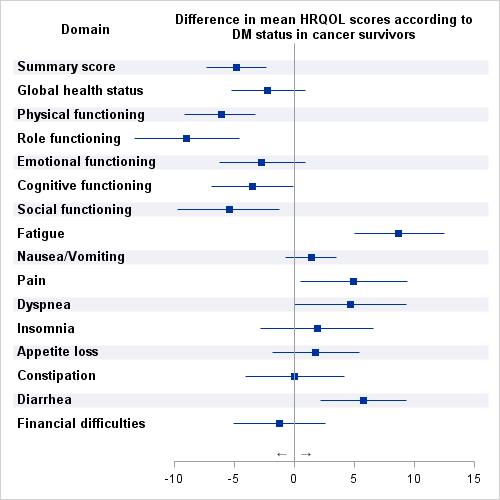


**c**

**b**

**a**

Figure S2. Difference in mean health related quality of life (HRQOL) scores between cancer survivors with and without diabetes mellitus (DM) at baseline, classified according to participation and vital status at follow-up (cross-sectional at baseline, n = 6,811)

a. Including cancer survivors who only responded at baseline and alive at follow-up (n = 2,854); b. Including cancer survivors who responded at both baseline and follow-up (n = 2,627); c. Including cancer survivors who deceased during follow-up (n = 1,330).

Footnote:

Reference group: participants without prevalent DM at baseline.

Results were adjusted for age at survey, age at cancer diagnosis, tumor-sex, education, socioeconomic deprivation, comorbidity status (the number of other comorbidities excluding DM), cancer stage, recurrence/metastasis, partnership, BMI, alcohol consumption, smoking status, physical activity.


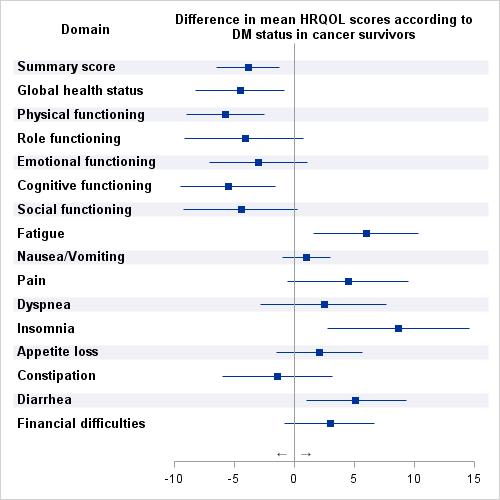

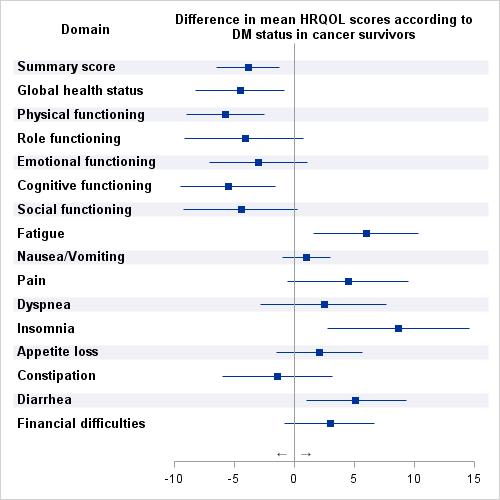

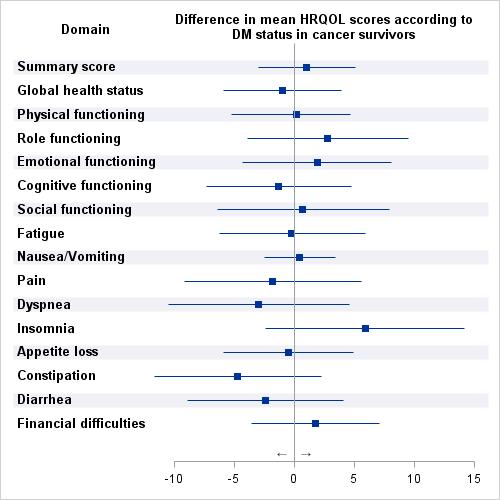


**c**

**b**

**a**

Figure S3. Difference in mean health related quality of life (HRQOL) scores among cancer survivors at follow-up according to diabetes mellitus (DM) status, classified according to the time of DM diagnosis (n = 2,627)

a. Prevalent DM (n = 254) vs. no DM (n = 2,252); b. Incident DM (n = 121) vs. no DM (n = 2,252); c. Incident DM (n = 121) vs. prevalent DM (n = 254).

Footnote:

Reference group: no DM at both baseline and follow-up.

Results were adjusted for age at survey, age at cancer diagnosis, tumor-sex, education, socioeconomic deprivation, comorbidity status (the number of other comorbidities excluding DM), cancer stage, recurrence/metastasis, partnership, BMI, alcohol consumption, smoking status, physical activity.


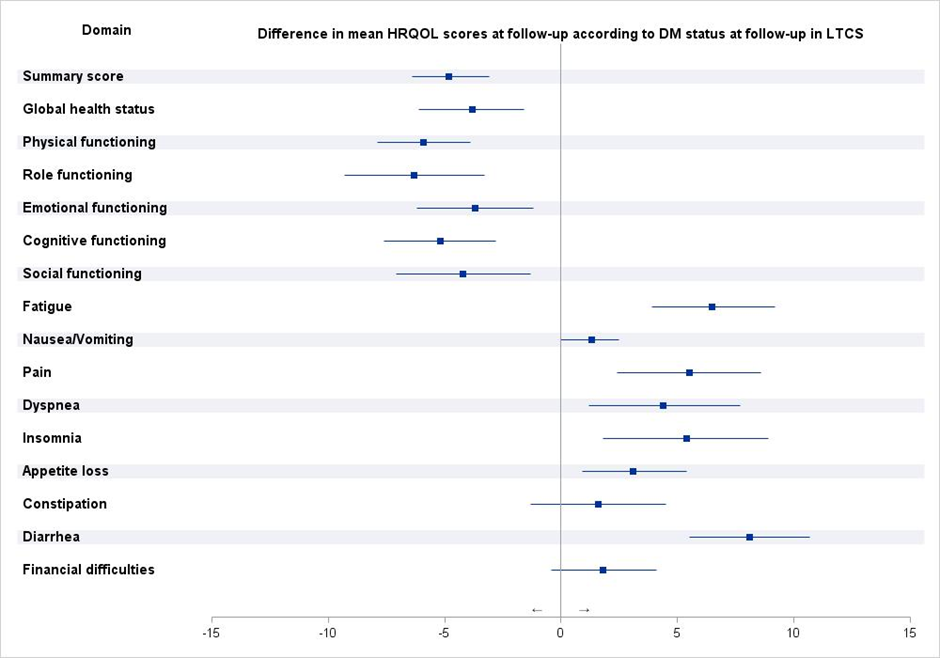


Figure S4. Difference in mean health-related quality of life (HRQOL) scores according to diabetes mellitus (DM) status in long-term cancer survivors (LTCS) at follow-up (cross-sectional at follow-up)

Footnote: difference in mean HRQOL scores and the 95% confidence intervals are displayed as forest plots (non-DM group served as the reference group). All results were adjusted for age at survey, age at cancer diagnosis, tumor-sex, education, socioeconomic deprivation, partnership, body mass index, alcohol consumption, smoking status, physical activity, comorbidity status (the number of other comorbidities excluding DM), cancer stage and recurrence/metastasis
